# Successful thrombectomy for inferior vena cava thrombosis associated with an ovarian tumor: a case report

**DOI:** 10.1093/jscr/rjaf196

**Published:** 2025-04-05

**Authors:** Kazuki Mori, Takashi Shuto, Kenshi Yoshimura, Shinji Miyamoto, Yusuke Sato, Mamiko Okamoto, Kentaro Kai, Eiji Kobayashi

**Affiliations:** Department of Cardiovascular Surgery, Oita University, 1-1 Idaigaoka, Hasama, Yufu, 879-5593, Japan; Department of Cardiovascular Surgery, Oita University, 1-1 Idaigaoka, Hasama, Yufu, 879-5593, Japan; Department of Cardiovascular Surgery, Oita University, 1-1 Idaigaoka, Hasama, Yufu, 879-5593, Japan; Department of Cardiovascular Surgery, Oita University, 1-1 Idaigaoka, Hasama, Yufu, 879-5593, Japan; Department of Obstetrics and Gynecology, Oita University, 1-1 Idaigaoka, Hasama, Yufu 879-5593, Japan; Department of Obstetrics and Gynecology, Oita University, 1-1 Idaigaoka, Hasama, Yufu 879-5593, Japan; Department of Obstetrics and Gynecology, Oita University, 1-1 Idaigaoka, Hasama, Yufu 879-5593, Japan; Department of Obstetrics and Gynecology, Oita University, 1-1 Idaigaoka, Hasama, Yufu 879-5593, Japan

**Keywords:** deep vein thrombosis, pulmonary embolism, inferior vena cava, thrombectomy, ovarian tumor

## Abstract

A 51-year-old female patient presented with abdominal distension. Computed tomography revealed a right ovarian tumor suspicious for clinical stage IC ovarian cancer, along with a considerable inferior vena cava (IVC) thrombus extending to the Th2 level, accompanied by a deep vein thrombus and a peripheral left pulmonary embolism (PE). Preoperative IVC filter placement was deemed unfavorable due to the short distance between the renal vein and the thrombus. On the second day of hospitalization, ovarian tumor extraction, thrombectomy, and IVC filter placement were performed. Pathology of the excised tumor revealed endometrioid carcinoma. Postoperatively, anticoagulation therapy with edoxaban 30 mg/day resulted in improvement of the deep vein thrombus and PE. The concomitant adnexal resection and thrombectomy rendered the safe placement of an IVC filter feasible despite the preoperative placement being unfeasible. Open thrombectomy remains a valid treatment strategy despite advances in endovascular therapy.

## Introduction

Malignancy is a known risk factor for deep vein thrombosis (DVT), which can lead to pulmonary embolism (PE) [1–3]. The surgical strategy for tumor resection with inferior vena cava (IVC) thrombus requires multidisciplinary planning and is of paramount importance. Our surgical team successfully performed simultaneous adnexal resection and open thrombectomy, followed by IVC filter placement. This case report contributes to the literature on the management of malignancy-associated venous thrombosis, particularly in the setting of ovarian cancer with extensive IVC thrombus.

## Case report

**Figure 1:**
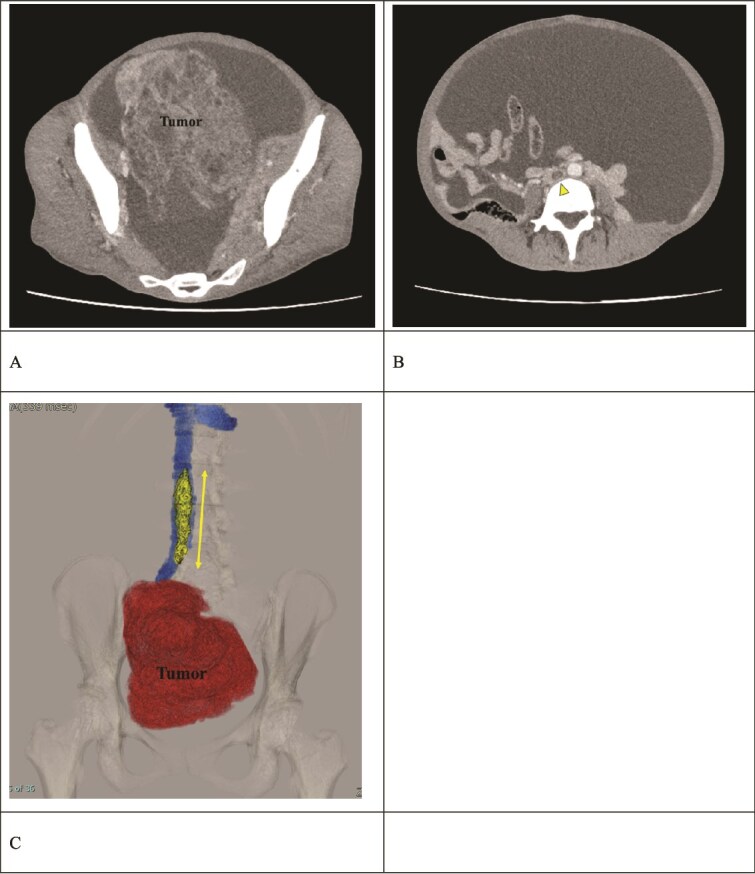
Preoperative contrast-enhanced CT. (A) A right ovarian tumor is observed in the pelvic cavity. (B) Massive ascites and a thrombus (arrow) in the IVC are observed. (C) The 3D reconstruction image shows a venous thrombus (arrow) extending from the right iliac vein to the IVC at the L2 level. The distance between the renal vein and the thrombus tip is only 25 mm.

A 51-year-old woman who has never been pregnant presented with abdominal bloating for the past 3 months. The patient’s serum CA125 and CA19-9 levels were significantly elevated (112 080 and 178.25 U/ml, respectively). Computed tomography (CT) revealed a pelvic mass consistent with a right ovarian tumor suspicious for ovarian cancer (clinical stage IC: T1a N0 N0), in addition to massive ascites (Fig. 1A); however, elevated D-dimer levels of 10.72 μg/ml were detected, and lower limb ultrasonography revealed bilateral DVT. Contrast-enhanced CT revealed a thrombus in the IVC extending from the right iliac vein to the L2 level, accompanied by a peripheral left PE (Fig. 1B and C). The patient had not been receiving any anticoagulant therapy. Following admission, the patient received continuous intravenous heparin infusion to prevent the progression of DVT and PE.

A multidisciplinary team consisting of gynecologists, cardiovascular surgeons, and radiologists concluded that preoperative IVC filter placement was contraindicated due to the short distance of only 25 mm between the renal vein and the thrombus tip. The surgical strategy was developed to consist of simultaneous adnexal resection and thrombectomy, followed by IVC filter placement subsequent to thrombus removal. On the second day of hospitalization, the patient underwent surgery because of the high risk of PE.

**Figure 2:**
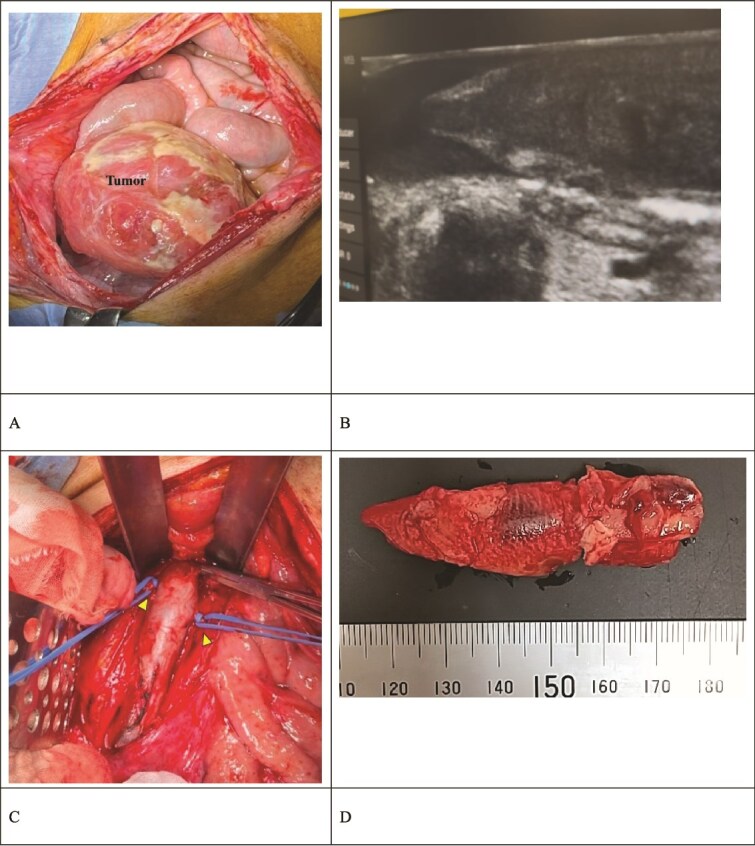
Intraoperative findings. (A) The ovarian tumor did not adhere to the peritoneal cavity. (B) Intraoperative ultrasound revealed a thrombus extending to the IVC below the renal vein. The thrombus had high echogenicity at the periphery and low echogenicity internally. (C) The IVC was clamped below the renal vein. The bilateral ovarian veins were ligated with vessel loops (arrow). (D) The extracted thrombus measured 65 × 15 mm and was already organized.

**Figure 3:**
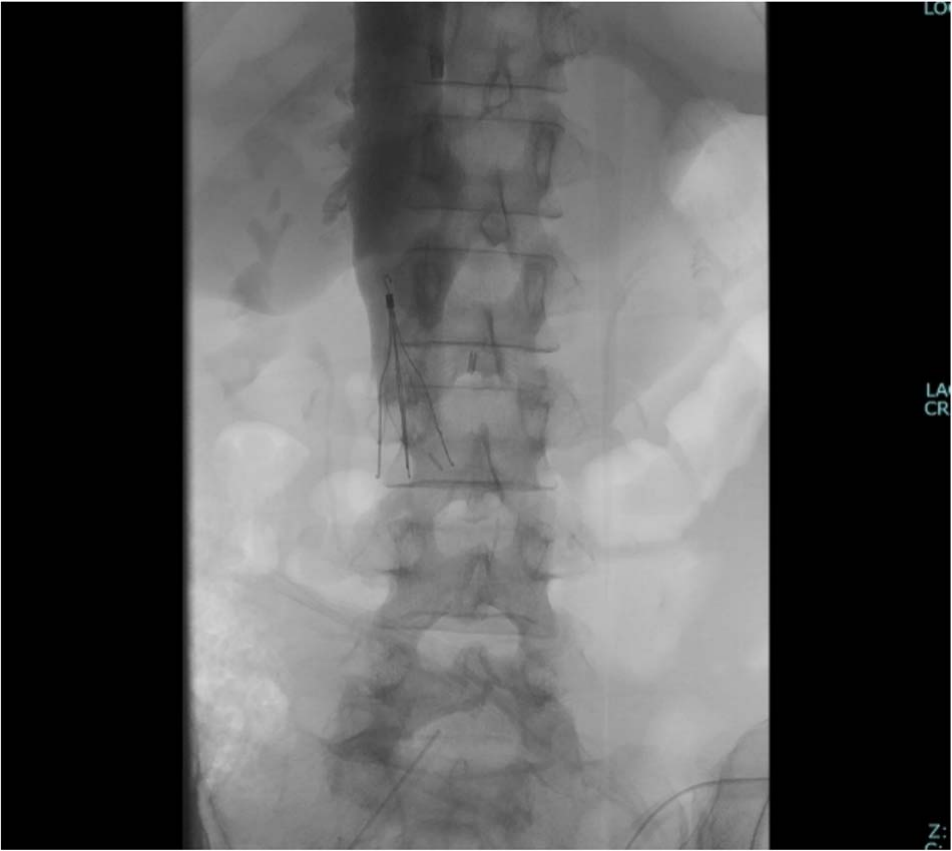
The IVC filter was placed after closing the abdomen.

The surgical procedure was conducted with extracorporeal membrane oxygenation available as a backup, accompanied by continuous intraoperative right heart transesophageal echocardiographic monitoring. The surgical approach was a midline laparotomy. The ovarian tumor was successfully excised as there were no intraabdominal adhesions (Fig. 2A). The IVC was carefully exposed after tumor resection, with care taken to prevent thrombus detachment. The extent of the thrombus was confirmed via ultrasonography (Fig. 2B). The bilateral ovarian veins were controlled with vessel loops, and the IVC was subsequently clamped below the renal veins (Fig. 2C). A venotomy was performed, and the thrombus was extracted (Fig. 2D). After confirming the absence of a residual thrombus through ultrasonography, the venotomy was closed using 5-0 polypropylene sutures. The IVC filter was placed via the jugular vein after the abdomen was closed (Fig. 3).

Pathological analyses of the excised tumor revealed an endometrioid carcinoma. No tumor component was identified within the thrombus. After surgery, anticoagulation was initiated with edoxaban 30 mg/day, and the patient was discharged on the 11th postoperative day without complications. A contrast-enhanced CT scan at 3 months postoperatively revealed an improvement in the DVT and PE. As malignancy contributes to a hypercoagulable state, long-term oral anticoagulation therapy is planned.

## Discussion

Gynecologic tumors are often complicated by DVT, which can result in life-threatening PE [1–3]. The malignancy-associated hypercoagulability, often termed Trousseau syndrome, can lead to a variety of thrombotic complications, including venous thromboembolism and arterial thrombosis [4, 5]. The patient’s condition was further complicated by massive ascites, which led to increased intraabdominal pressure and consequently, venous return impairment. This, in addition to the malignancy-induced hypercoagulable state, made the patient more susceptible to thrombus formation [6–8].

Preoperative anticoagulation therapy has been demonstrated to achieve successful thrombus dissolution in cases of acute thrombosis [3, 9]. In this case, the size of the thrombus raised concerns about the risk of a massive PE, which could have been fatal. Therefore, it was determined that an urgent surgical intervention was necessary. Intraoperatively, the thrombus was found to be organized, indicating that it was unlikely to respond to anticoagulation therapy.

Tumor excision, while providing relief from venous compression, may result in an increase in venous return, thereby raising concerns about the potential detachment of the attached thrombus. A prevalent approach involves the initiation of preoperative anticoagulation, after which IVC filters are inserted prior to tumor resection [3, 7]. However, in this case, the limited distance between the renal vein and the thrombus precluded preoperative IVC filter placement. Subsequent to the thrombectomy, an IVC filter was successfully implanted to manage the persistent bilateral lower extremity DVT. Even with preoperative IVC filter placement, thrombectomy would likely have been necessary due to the size of the IVC thrombus.

IVC filter placement can be technically difficult when the thrombus extends to a high level within the IVC or when the tumor exerts significant pressure on the former [8]. There are some reports suggesting that IVC occlusion, which is achieved by inserting an IVC catheter through the jugular vein, can be a viable PE prevention strategy [8, 10, 11]. In this case, the central pulmonary artery balloon occlusion procedure was not employed; however, it is a viable option for intraoperative PE prophylaxis when preoperative IVC filter placement is not feasible. Hemodynamic instability should be considered when performing high IVC balloon occlusion [11]. Recently developed endovascular thrombectomy systems provide a valuable treatment option [12].

Despite the successful completion of the surgical procedure without intraoperative thrombus detachment, the significant risk of a major intraoperative PE required meticulous planning and execution in close coordination with the anesthesiology and cardiovascular surgery teams [7].

In conclusion, the sequence of thrombectomy and the timing of IVC filter placement should be individualized based on a comprehensive assessment of each patient’s unique clinical presentation and risk profile. This case highlights that even in the era of widespread endovascular intervention, open thrombectomy remains a vital approach in managing complex venous thromboembolism associated with gynecological malignancies.
